# Supplementary material for: Type I interferons affect the metabolic fitness of CD8+ T cells from patients with systemic lupus erythematosus
Source: Nat Commun. 2021 Mar 31;12:1980. doi: 10.1038/s41467-021-22312-y (PMC8012390; doi:10.1038/s41467-021-22312-y)
Supplement: Supplementary file 1 — Supplementary Information [file 41467_2021_22312_MOESM1_ESM.pdf]

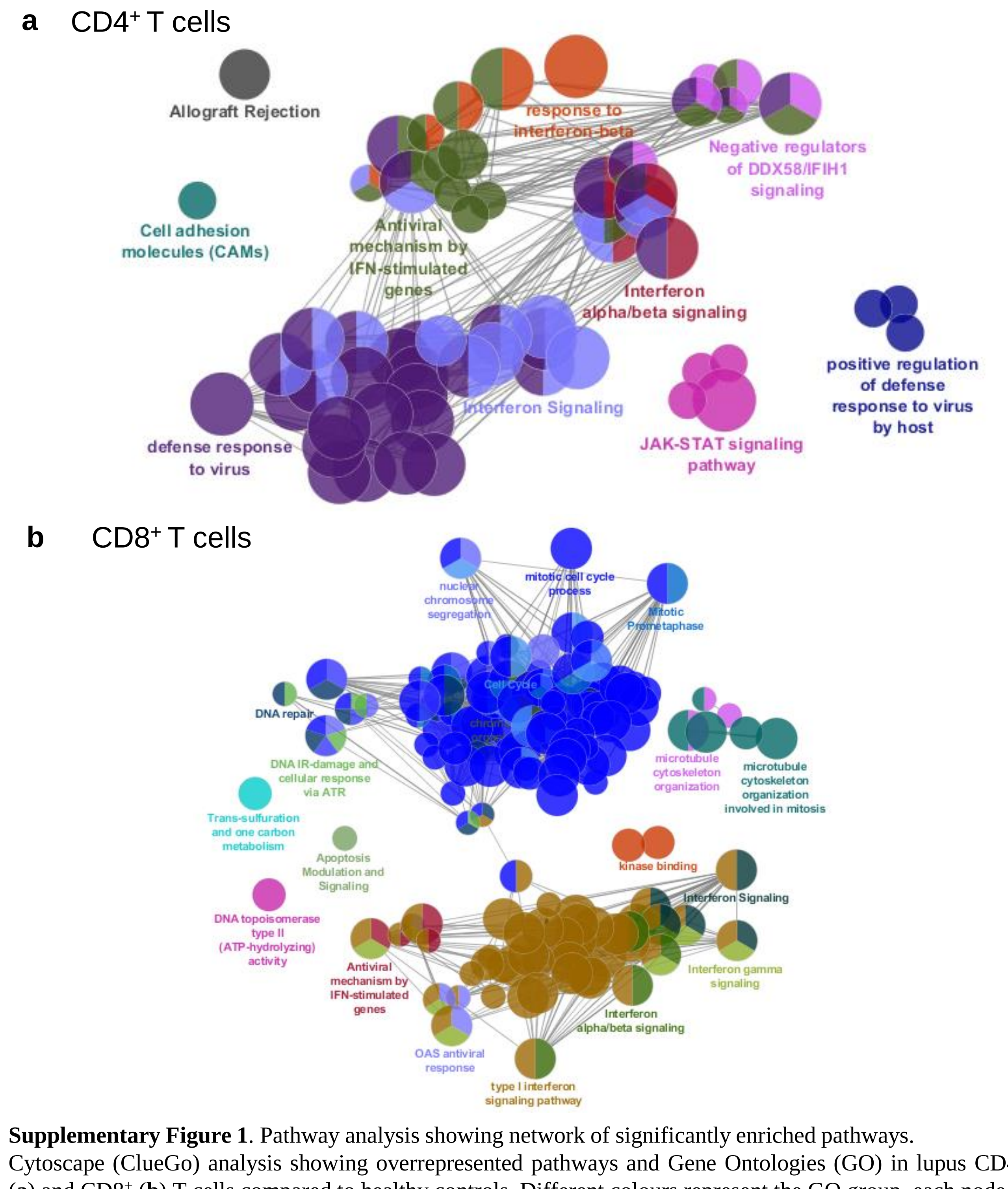


**Supplementary Figure 1**. Pathway analysis showing network of significantly enriched pathways. Cytoscape (ClueGo) analysis showing overrepresented pathways and Gene Ontologies (GO) in lupus CD4$^+$ (**a**) and CD8$^+$ (**b**) T cells compared to healthy controls. Different colours represent the GO group, each node is a pathway/GO and the edges show the connectivity between each node based on the number of genes shared by two given pathways. The size of the nodes represents the number of genes on each pathway/GO. Differential expressed genes were analysed with Cytoscape ClueGo v.2.3.3. (http://apps.cytoscape.org/apps/cluego) based of GO (Biological Processes, Molecular Functions, Immune System Process), Interpro, KEGG, Reactome and Wiki Pathways. Go Term Fusion and grouping were performed. Terms were called enriched based on maximum P value of 0.05 and a minimum of 3% overlap. To correct the P-values for multiple testing, Bonferroni step-down was performed. Enriched groups were further ranked according to the group, Bonferroni's step-down-adjusted P value. Group lead terms were defined inside each group as the term with the lowest adjusted P value of enrichment.

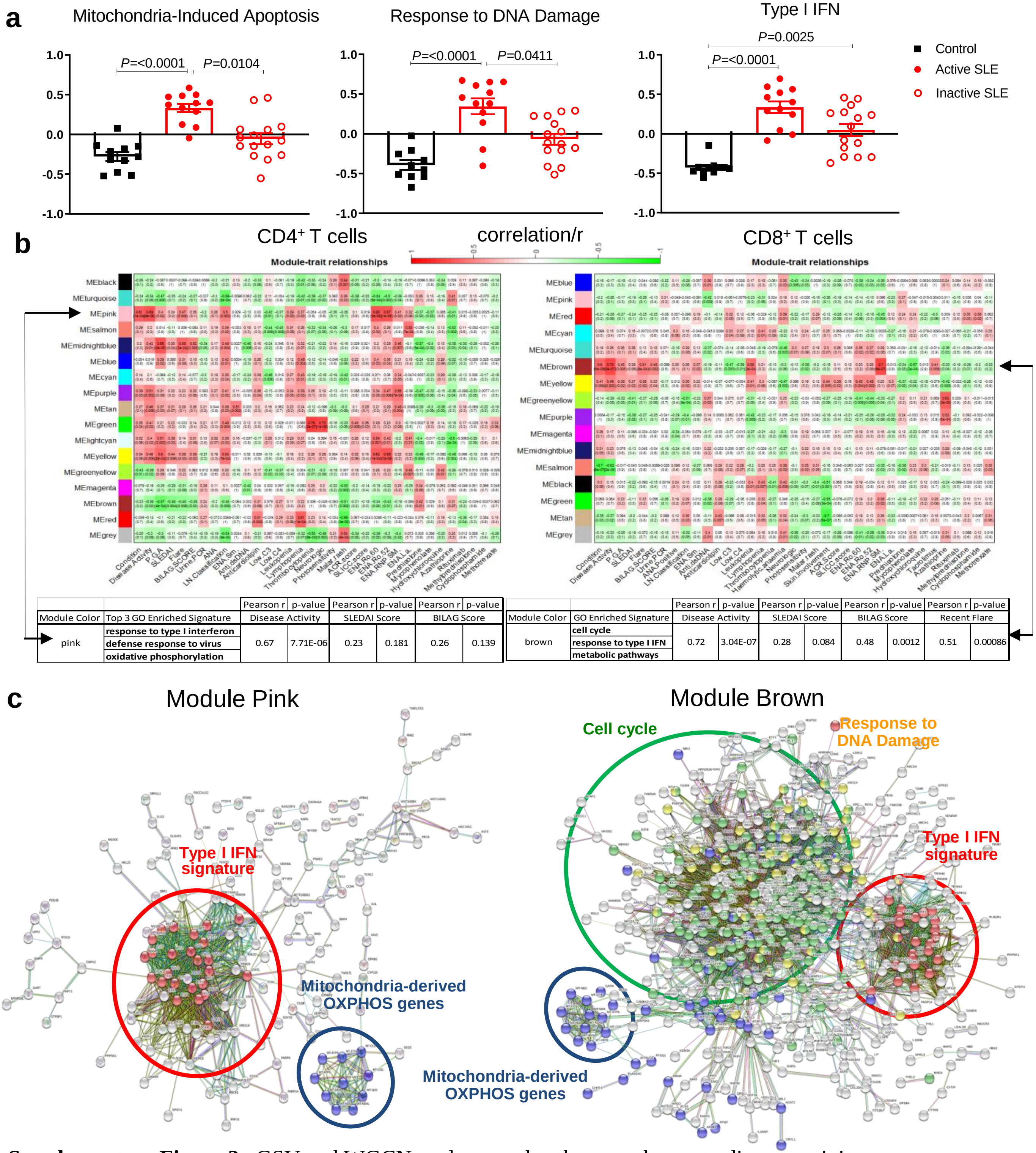


| Module Color | Top 3 GO Enriched Signature | Disease Activity Pearson r | Disease Activity p-value | SLEDAI Score Pearson r | SLEDAI Score p-value | BILAG Score Pearson r | BILAG Score p-value |
|---|---|---|---|---|---|---|---|
| pink | response to type I interferon<br>defense response to virus<br>oxidative phosphorylation | 0.67 | 7.71E-06 | 0.23 | 0.181 | 0.26 | 0.139 |

| Module Color | GO Enriched Signature | Disease Activity Pearson r | Disease Activity p-value | SLEDAI Score Pearson r | SLEDAI Score p-value | BILAG Score Pearson r | BILAG Score p-value | Recent Flare Pearson r | Recent Flare p-value |
|---|---|---|---|---|---|---|---|---|---|
| brown | cell cycle<br>response to type I IFN<br>metabolic pathways | 0.72 | 3.04E-07 | 0.28 | 0.084 | 0.48 | 0.0012 | 0.51 | 0.00086 |

**Supplementary Figure 2** . GSV and WGCN analyses and pathways relevant to disease activity

(**a**) GSVA of CD8$^{+}$ T cell transcriptomes between active vs inactive SLE patients. Enrichment scores calculated for DNA damage, mitochondria-induced apoptosis and type I IFN gene sets are shown. Data presented as mean ± S.E.M. Each symbol represents an individual. (HC n=10; Active SLE n=12; Inactive SLE n=15). Kruskal-Wallis non-parametric test; Dunn's Multiple Comparison Test; only significant differences are indicated. Source data for this figure are provided as a Source Data file. (**b**) Heatmaps illustrating the correlation of co-expression modules (coloured blocks, y axis) derived from CD4$^{+}$ and CD8$^{+}$ T cell transcriptomes of 28 SLE patients with clinical traits (x axis). Pearson correlation, r, and p-value are shown for module pink and brown. (**c**) Network of co-expressing genes derived by STRING (high confidence score = 0.7) (https://string-db.org/) for all genes in module pink and brown, illustrating genes corresponding to type I IFN response and mitochondria-derived oxidative phosphorylation (OXPHOS) genes.

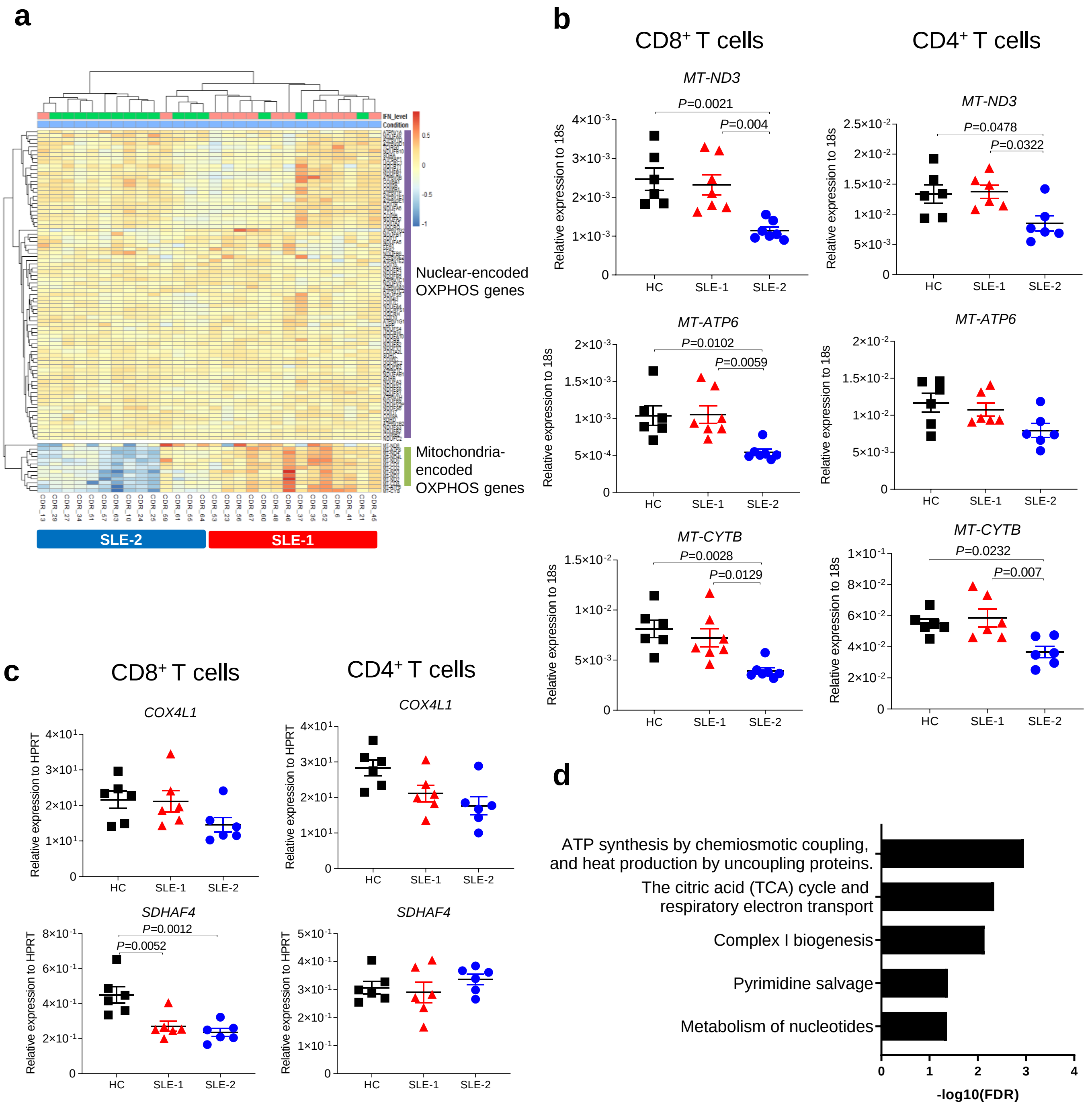


**Supplementary Figure 3.** Differential expression analysis between SLE-1 and SLE-2 groups. (**a**) Heatmap of all genes involved in 'Oxidative Phosphorylation' according to KEGG pathway database. RT-PCR of (**b**) mitochondria-derived (HC n=6; SLE-1 n=6-7; SLE-2 n=6-7), and (**c**) nucleus-derived genes involved in OXPHOS in isolated CD4$^+$ and CD8$^+$ T cells from SLE patients in group 1 and 2 and controls. (HC n=6; SLE-1 n=6; SLE-2 n=6) (**D**) Significantly enriched metabolic pathways downregulated in SLE-2 compared to SLE-1 patients. Metabolic pathway enrichment was performed with Cytoscape Reactome Functional Interaction app. Pathways with a maximum false discovery rate of 0.05 were retained. (**b-c**) Data presented as mean ± S.E.M. Each symbol represents one individual. One-way ANOVA, only significant differences are indicated. HC=healthy control; FDR= fold discovery rate. Source data for this figure are provided as a Source Data file.

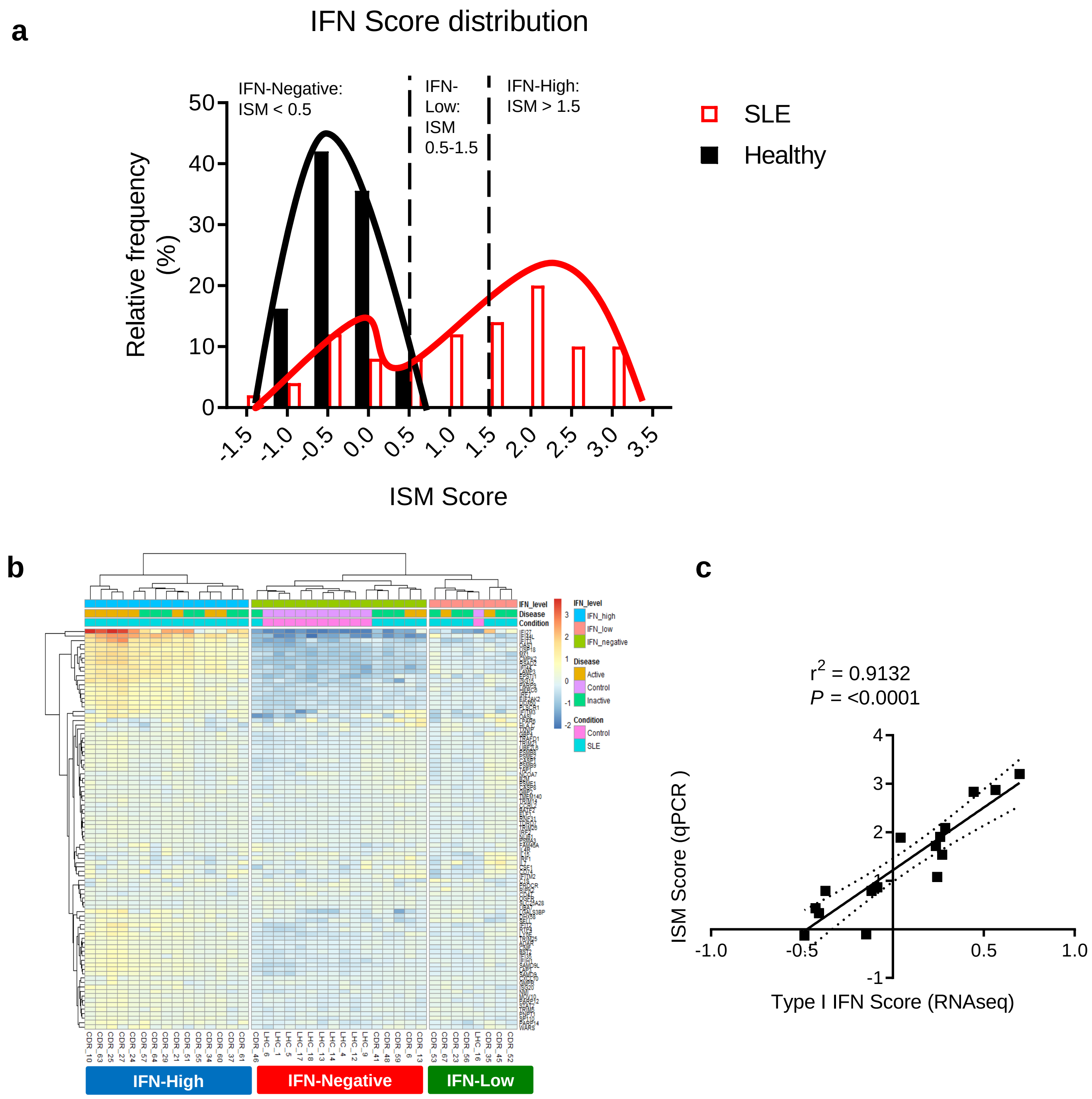


**Supplementary Figure 4.** IFN Score Matrix (ISM)
(**a**) Distribution of ISM values in healthy controls (black bar) and SLE patients (red bar). The ISM distribution among SLE patients is bimodal. The dashed lines indicate cut-off used for IFN-Neg (ISM < 0.5), IFN-Low (ISM 0.5-1.5) and IFN-High (ISM > 1.5) patients. SLE n=50, healthy controls n=31. (**b**) Unsupervised hierarchical clustering of 97 ISGs in CD8$^{+}$ T cells. Three groups were identified: high ISG expression (active n=8, inactive n=7); low ISG expression (active n=2, inactive n=5, healthy control=1); no ISG expression (active n=2, inactive n=4, healthy control=10). (**c**) Pearson correlation, $r^2$, ($r^2$ = 0.9132. p-value <0.0001) of qPCR-based ISM score with RNAseq-derived score (n=16). Source data for this figure are provided as a Source Data file.

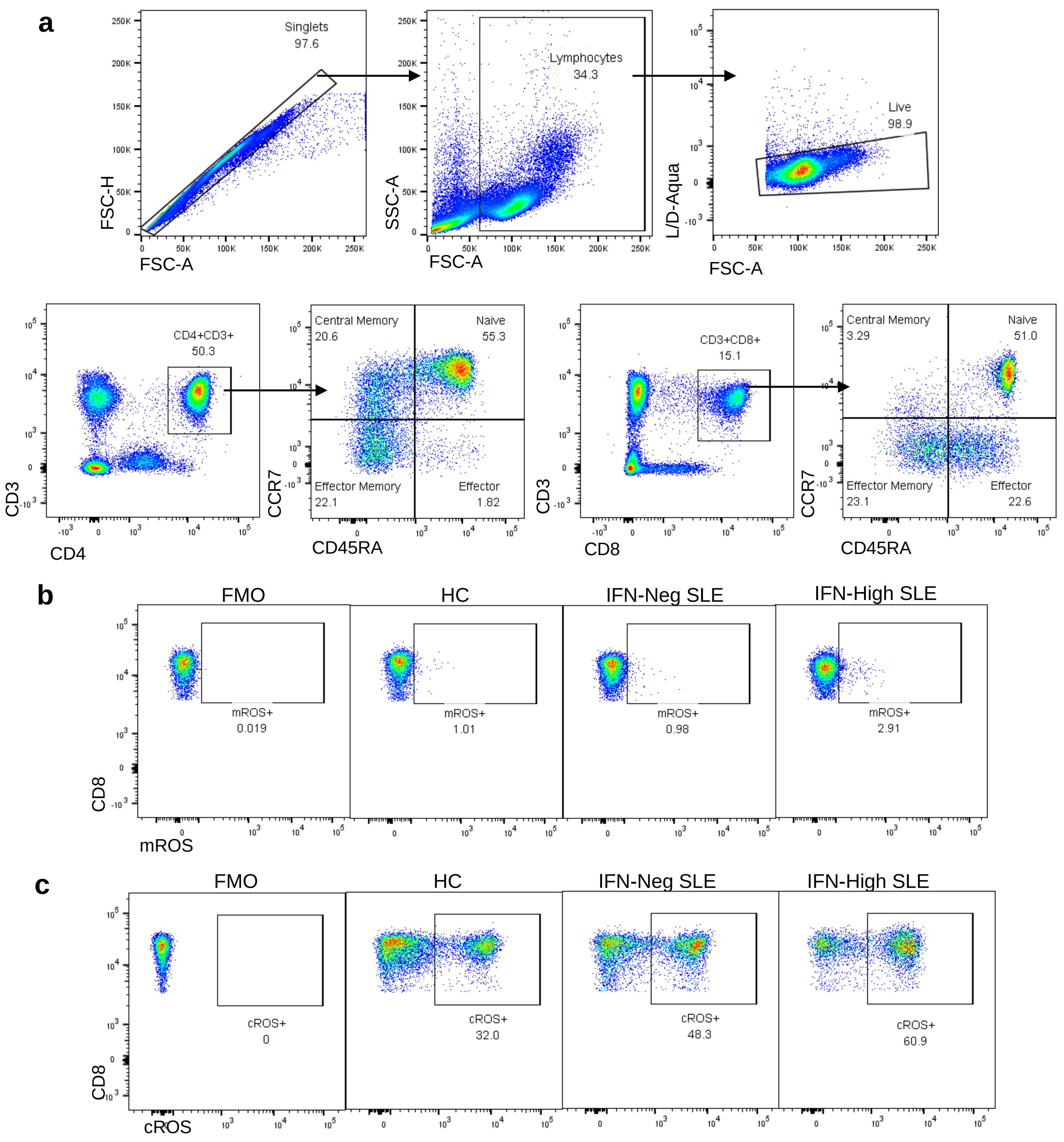


**Supplementary Figure 5** Representative gating strategies for T cell subsets and ROS$^+$ cells .
(**a**) PBMCs were stained with LD-Aqua followed by surface markers CD3, CD4, CD8, CCR7, and CD45RA antibodies. Duplets and cell debris were removed based on FCS/SSC and only live cells (LD-Aqua negative) population was analysed. T cell subsets were defined by CCR7 and CD45RA surface expression. (**b-c**) Gated CD8$^+$ T cell. Representative gating strategies for MitoSOX™ Red (mROS) positive cells (**b**) and cellROX Deep Red (cROS) positive cells (**c**) were shown. HC=healthy control; FMO=Fluorescence Minus One control. Data in Figure 2 (a-d), Supplementary Figure 6 and 7 were generated using these gating strategies. When mentioned, MitoTracker green (MTG); membrane potential dependent-Mitotracker Deep Read (MTDR); or Tetramethylrhodamine (TMRM) staining was added into the panel and MFI is showed.

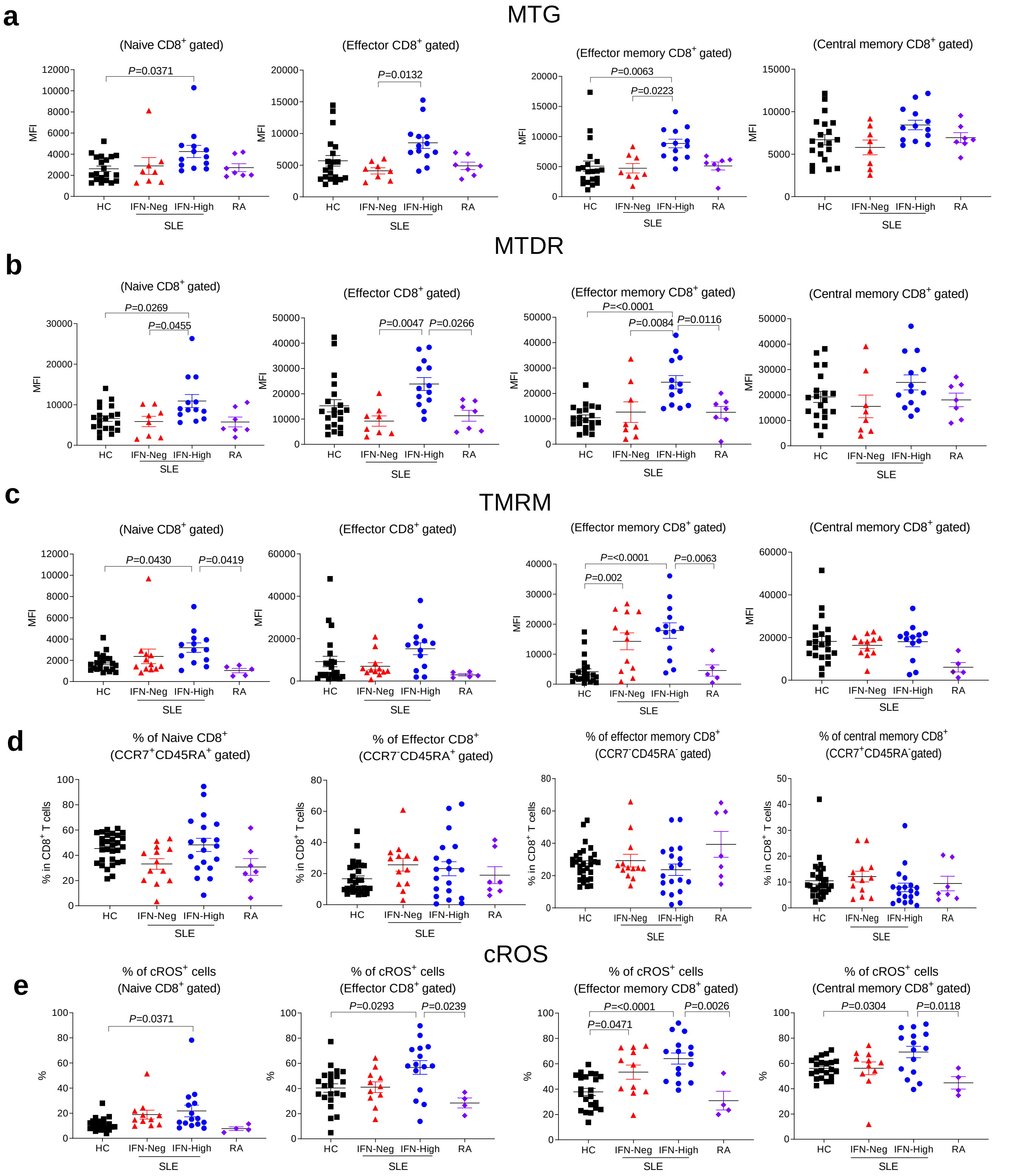


**Supplementary Figure 6** Assessment of mitochondrial phenotype in $CD8^+$ T cell subpopulations. $CD8^+$ T cell subsets were defined by CCR7 and CD45RA surface expression. Gated $CD8^+$ T cell subpopulations were stained with (**a**) MitoTracker green (MTG) (HC n=20; IFN-Neg n=8; IFN-High n=13; RA n=7).; (**b**) membrane potential dependent-Mitotracker Deep Read (MTDR) (HC n=20; IFN-Neg n=8; IFN-High n=13; RA n=7).; (**c**) Tetramethylrhodamine (TMRM). Mean fluorescence intensity (MFI) data are presented. (HC n=22; IFN-Neg n=12; IFN-High n=13; RA n=5).; (**d**) Proportion of each $CD8^+$ T subset (gated total $CD8^+$ T cells). (HC n=30; IFN-Neg n=13; IFN-High n=19; RA n=7). (**e**) Proportions of cellROX Deep Red (cROS) positive cells are shown. (HC n=22; IFN-Neg n=11; IFN-High n=15; RA n=4). (**a-e**) Data presented as mean ± S.E.M. Each symbol represents an individual. One-way ANOVA, only significant differences are indicated; HC=healthy control; RA=Rheumatoid Arthritis patients. Source data for this figure are provided as a Source Data file

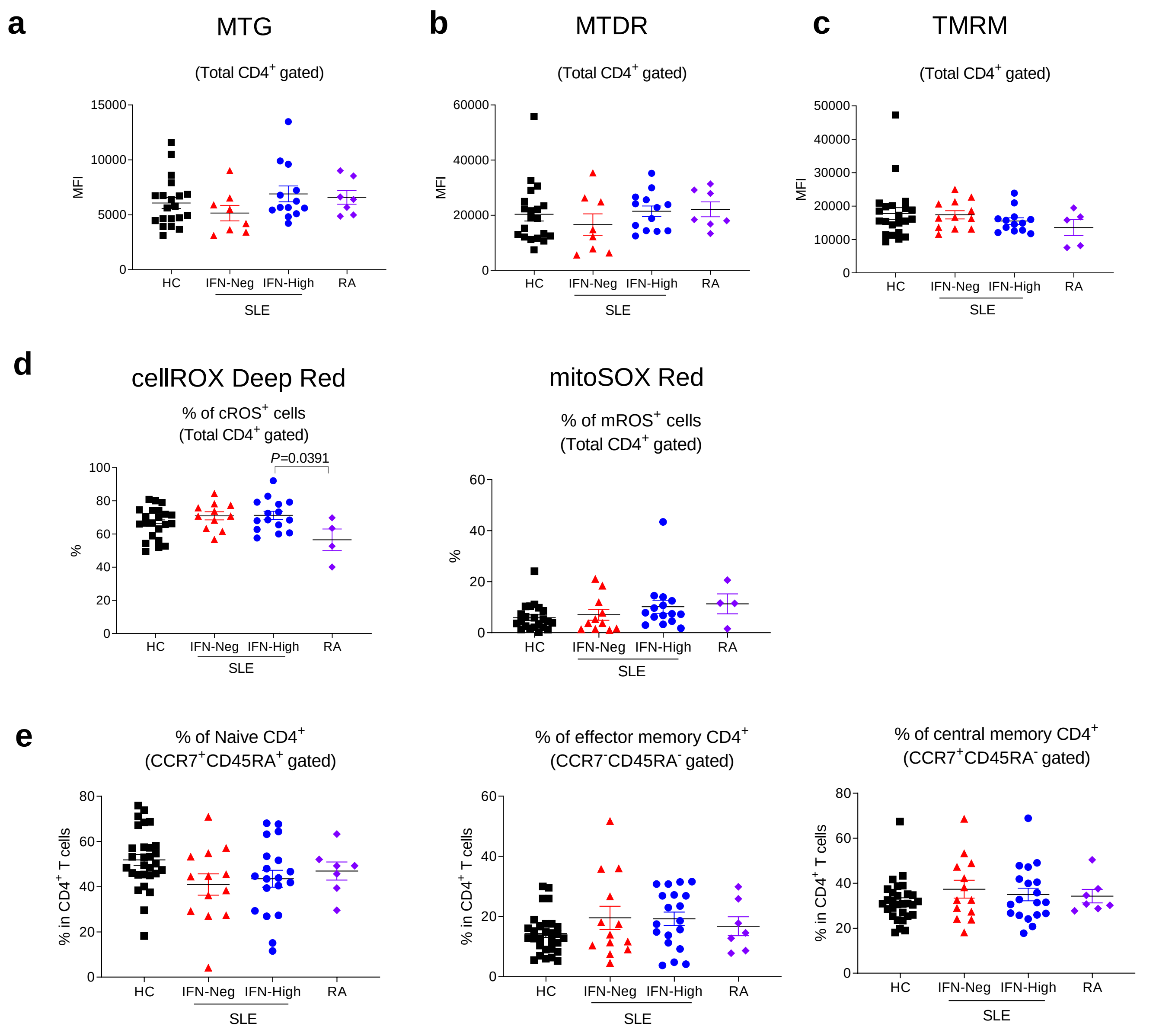


**Supplementary Figure 7**. Mitochondrial changes in total CD4+ T cells from lupus patients.
Gated CD4+ T cells were stained with (**a**) MitoTracker green (MTG) (HC n=20; IFN-Neg n=8; IFN-High n=13; RA n=7); (**b**) membrane potential dependent-Mitotracker Deep Read (MTDR) (HC n=20; IFN-Neg n=8; IFN-High n=13; RA n=7); (**c**) Tetramethylrhodamine (TMRM). (HC n=22; IFN-Neg n=12; IFN-High n=13; RA n=5); Mean fluorescence intensity (MFI) data are shown. (**d**) Proportions of CD4+ T cells positive for cROS (cellROX® Deep Red) and mROS (MitoSOX™ Red) are shown. (HC n=22; IFN-Neg n=11; IFN-High n=15; RA n=4); (**e**) Proportion of CD4+ T cell subsets defined by CCR7 and CD45RA surface expression. (HC n=29; IFN-Neg n=13; IFN-High n=19; RA n=7). (**a-e**) Data presented as mean ± S.E.M. Each symbol represents an individual. One-way ANOVA, only significant differences are indicated. HC=healthy controls; RA=Rheumatoid Arthritis patients.. Source data are for this figure provided as a Source Data file

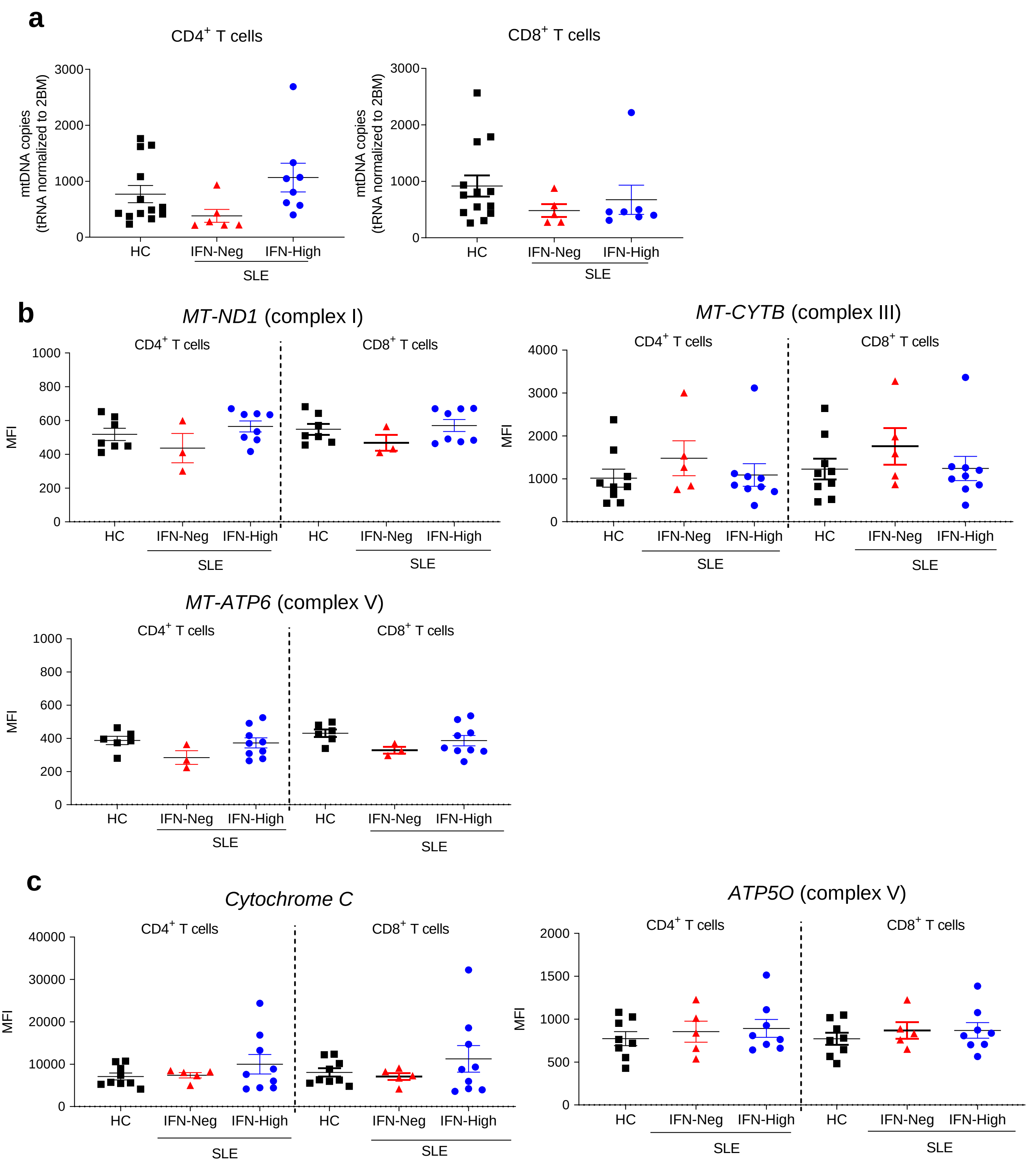


**Supplementary Figure 8** Mitochondria DNA copies and protein expression in T cells. (**a**) Mitochondria DNA copy number assessed by RT-PCR (HC n=13; IFN-Neg n=5-6; IFN-High n=7). Protein expression of (**b**) mDNA-encoded (HC n=6-9; IFN-Neg n=3-5; IFN-High n=8-9) and (**c**) nDNA-encoded OXPHOS genes measured by flow cytometry (HC n=8-9; IFN-Neg n=5; IFN-High n=8-9). Data presented as mean ± S.E.M. Each symbol represents an individual. One-way ANOVA, only significant differences are indicated. MFI=Mean fluorescence intensity; MT-ND1=Mitochondrially Encoded NADH:Ubiquinone Oxidoreductase Core Subunit 1; MT-ATP6=Mitochondrially Encoded ATP Synthase Membrane Subunit 6; MT-CYTB=Mitochondrially Encoded Cytochrome B; ATP5O=ATP Synthase Peripheral Stalk Subunit OSCP. Source data for this figure are provided as a Source Data file

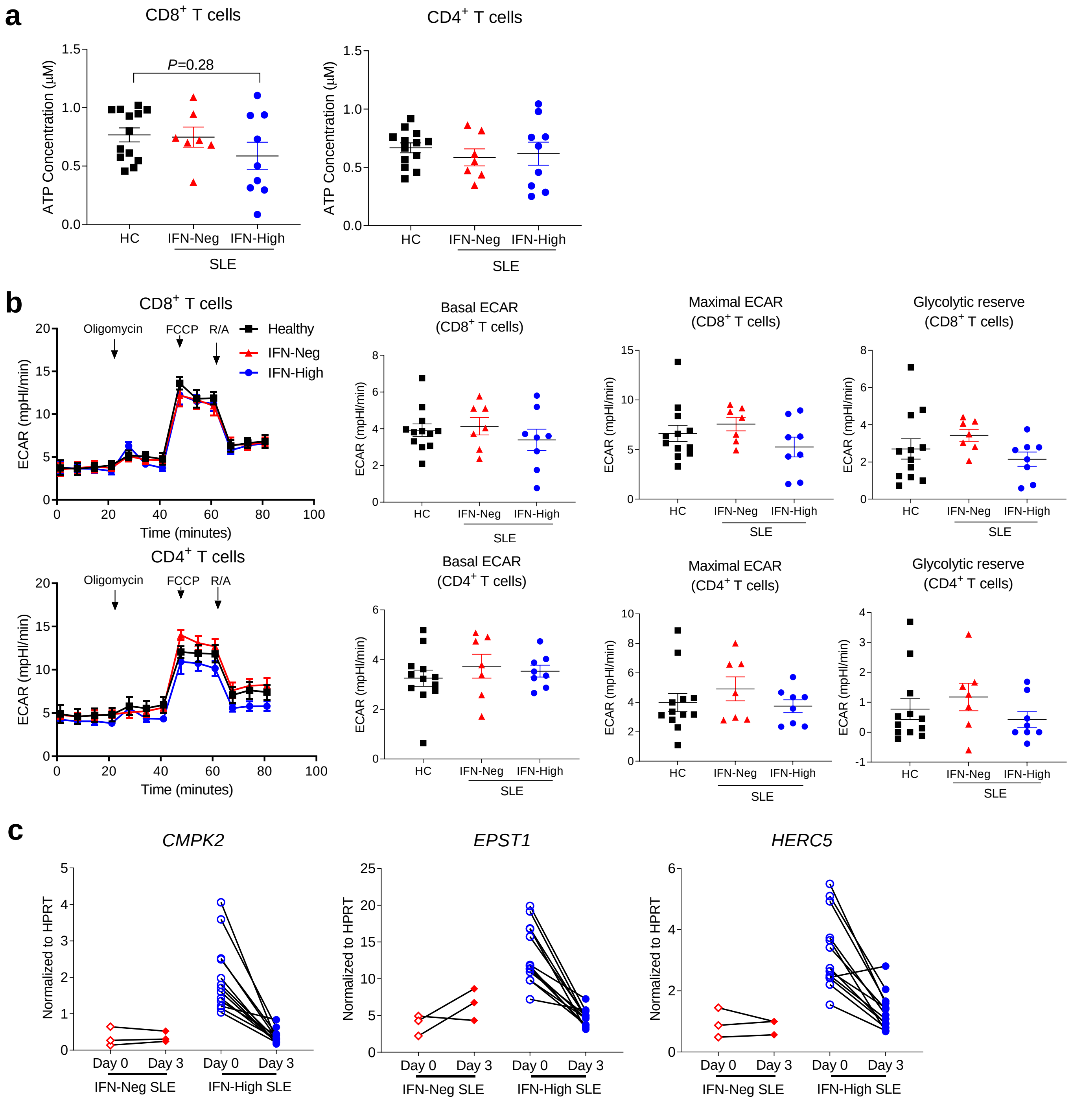


**Supplementary Figure 9** Metabolic analysis and IFN-inducible transcript changes *in vitro*
(**a**) Intracellular ATP concentrations in CD4$^+$ and CD8$^+$ T cells. Each symbol represents an individual:(HC n=7-13; IFN-Neg n=3-6; IFN-High n=8-9). (**b**) Representative extracellular acidification rate (ECAR) graphs (left panels) and summary graphs (right panels) of sorted CD4$^+$ T cells from controls, IFN-Neg and IFN-High lupus patients during a mitochondrial stress test. Oligomycin, carbonylcyanide p-trifluoromethoxyphenylhydrazone (FCCP), and rotenone/antimycin A (R/A) were added to the cells as indicated. Each symbol represents an individual: HC n=12; IFN-Neg n=7; IFN-High n= 7-8. (**c**) Expression analysis of IFN-inducible transcripts in IFN-Neg (n=3) and IFN-High (n=13) SLE CD8$^+$ T cells *ex vivo* and after 3 days in culture. **(A-B)** Data presented as mean ± S.E.M. One-way ANOVA, only significant differences are indicated; HC=healthy control. Source data for this figure are provided as a Source Data file

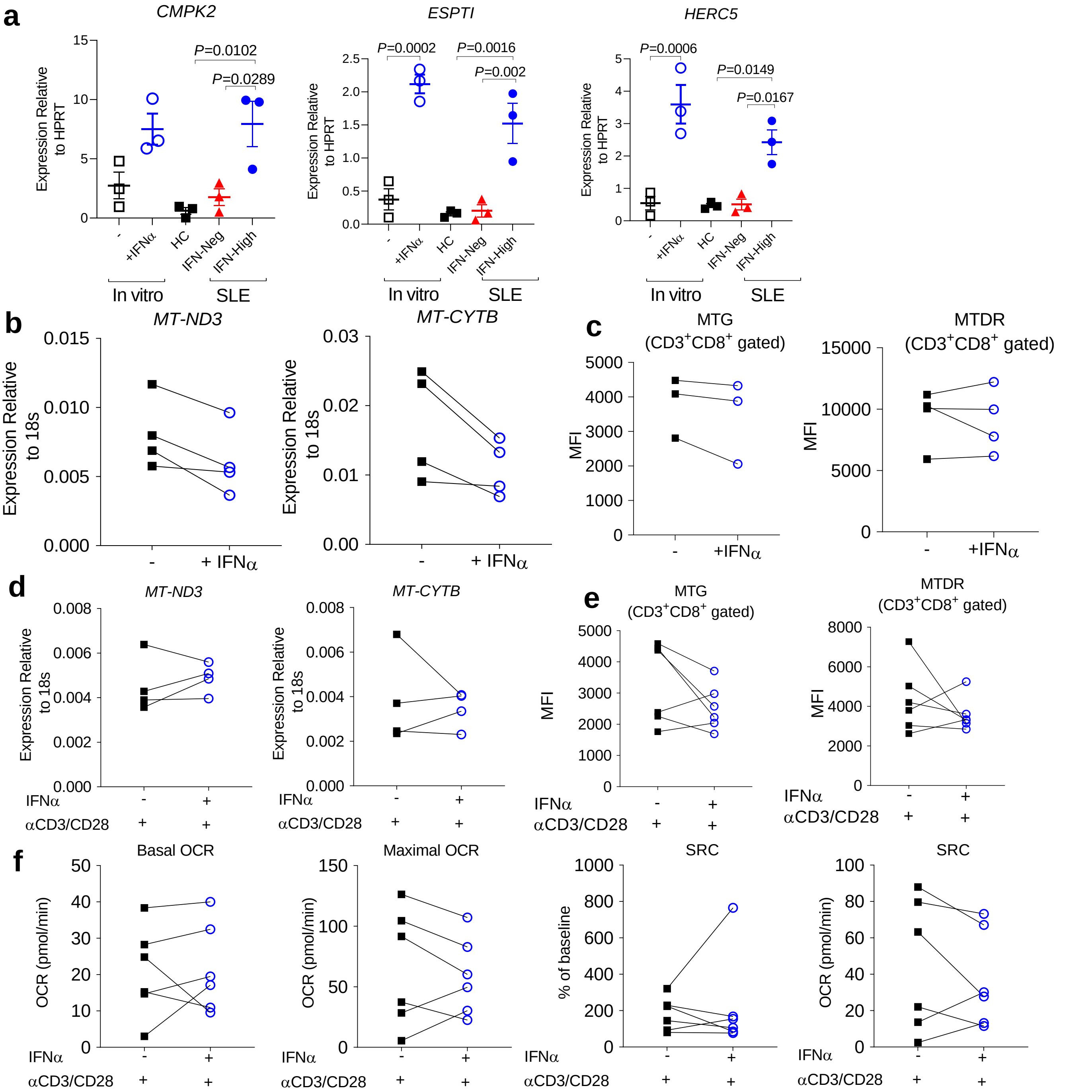


**Supplementary Figure 10.** ISG expression and metabolic changes after 2-day IFNα exposure.
**(a)** PBMCs from HC were treated with IL-2 (10U/ml) with or without 1000U/ml IFNα for 7 days and ISG expression measured using qPCR. ISG expression level in PBMCs treated with IFN-α, from IFN-Neg and IFN-High SLE patients and untreated HC (n=3 for each group). **(b-e)** Mitochondria-encoded gene expression (n=4 for each group) **(b** and **d)** and mitochondrial phenotype (n=3-6 for each group) **(c** and **e)** of purified CD8$^+$ T cells after 2 days treatment with IFNα (1000U/ml) alone **(b-c)** or with CD3/CD28 beads stimulation with or without IFNα **(d-e)**. Mitotracker Green (MTG) and Mitotracker Deep Red (MTDR) staining; Mean fluorescence intensity (MFI) are shown; gating was performed on CD3$^+$CD8$^+$ cells. **(f)** Oxidative capacities of sorted CD8$^+$ T cells from PBMCs treated as in **(d** and **f)**. Each dot represents one donor (n=6 for each group). (**a-f**) Data presented as mean ± S.E.M. **(a)** One-way ANOVA, **(b-f)** Two-tailed Wilcoxon matched-pairs signed rank test; only significant differences are indicated. *MT-ND3*=Mitochondrially Encoded NADH:Ubiquinone Oxidoreductase Core Subunit 3; *MT-CYTB*=Mitochondrially Encoded Cytochrome B; *HERC5*= HECT and RLD Domain Containing E3 Ubiquitin Protein Ligase 5; *EPST1*=epithelial stromal interaction 1; *CMPK2*=Cytidine/Uridine Monophosphate Kinase 2; HC=healthy control.. Source data for this figure are provided as a Source Data file.

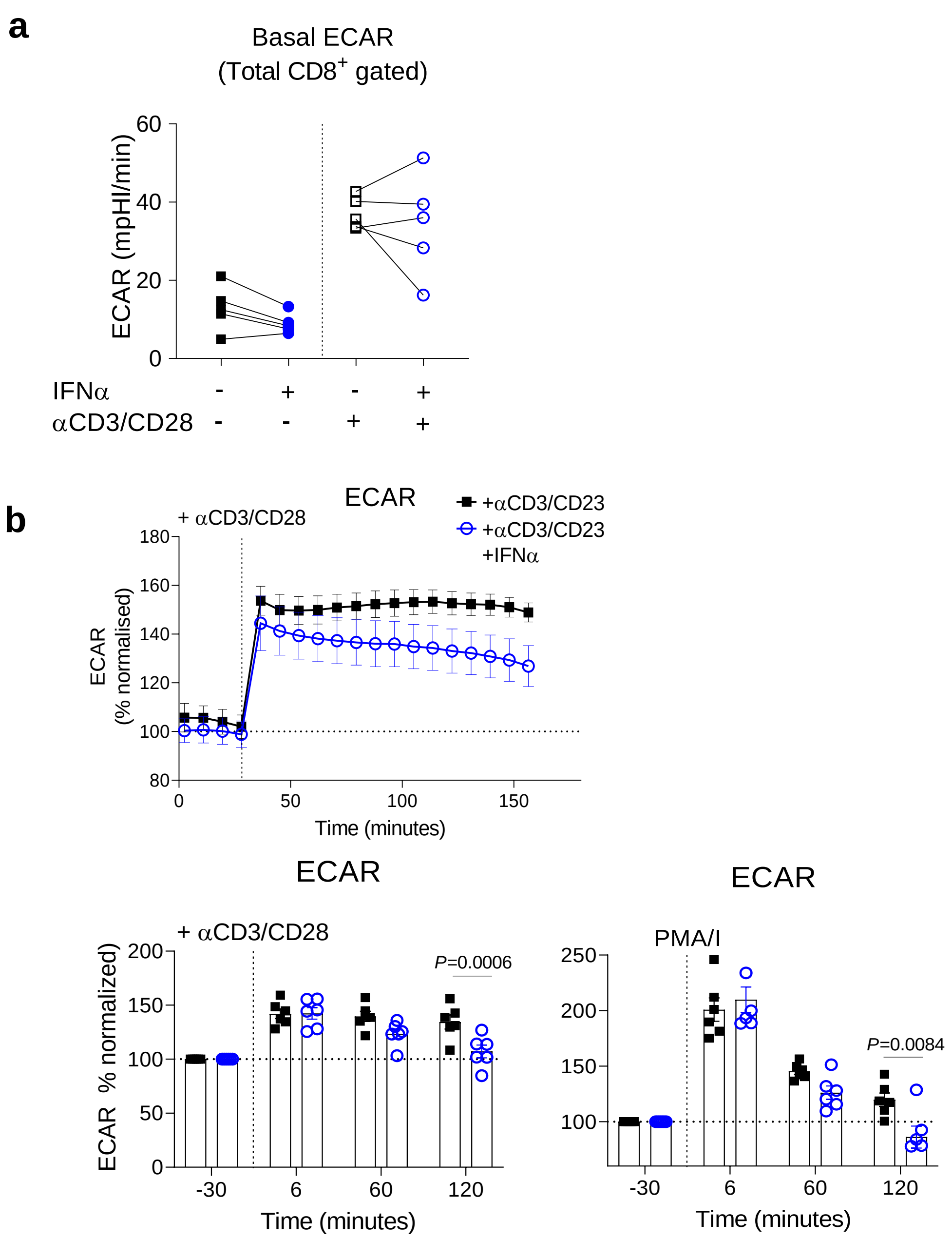


**Supplementary Figure 11.** Glycolysis response upon 7-day IFNα exposure and T cell activation

**(a-b)** PBMC from healthy donors treated with or without αCD3/CD28 beads in the presence or absence of 1000U/ml IFNα for 7 days. **(a)** Metabolic analyses of cell-sorted CD8+ T cells using the extracellular flux assay. Summary graph of basal glycolysis is shown. Each symbol represents one donor (n=5). (**b**) ECAR levels in cell-sorted CD8+ T cells upon re-stimulation with anti-CD3/CD28 beads or PMA/I injected during the extracellular flux assay. After 7 days in culture, αCD3/CD28 beads were removed and the cells were rested overnight prior to re-activation. A representative graph (top panel) and levels (bottom panels) at different time points normalised to basal level of each individual are shown. Data presented as mean ± S.E.M. Each symbol represents one donor (n=6). **(a)** Two-tailed Wilcoxon matched-pairs signed rank test was utilized; **(b)** two-way ANOVA; only significant differences are indicated. PMA/I=phorbol12-myristate13-acetate/Ionomycin; Source data for this figure are provided as a Source Data file

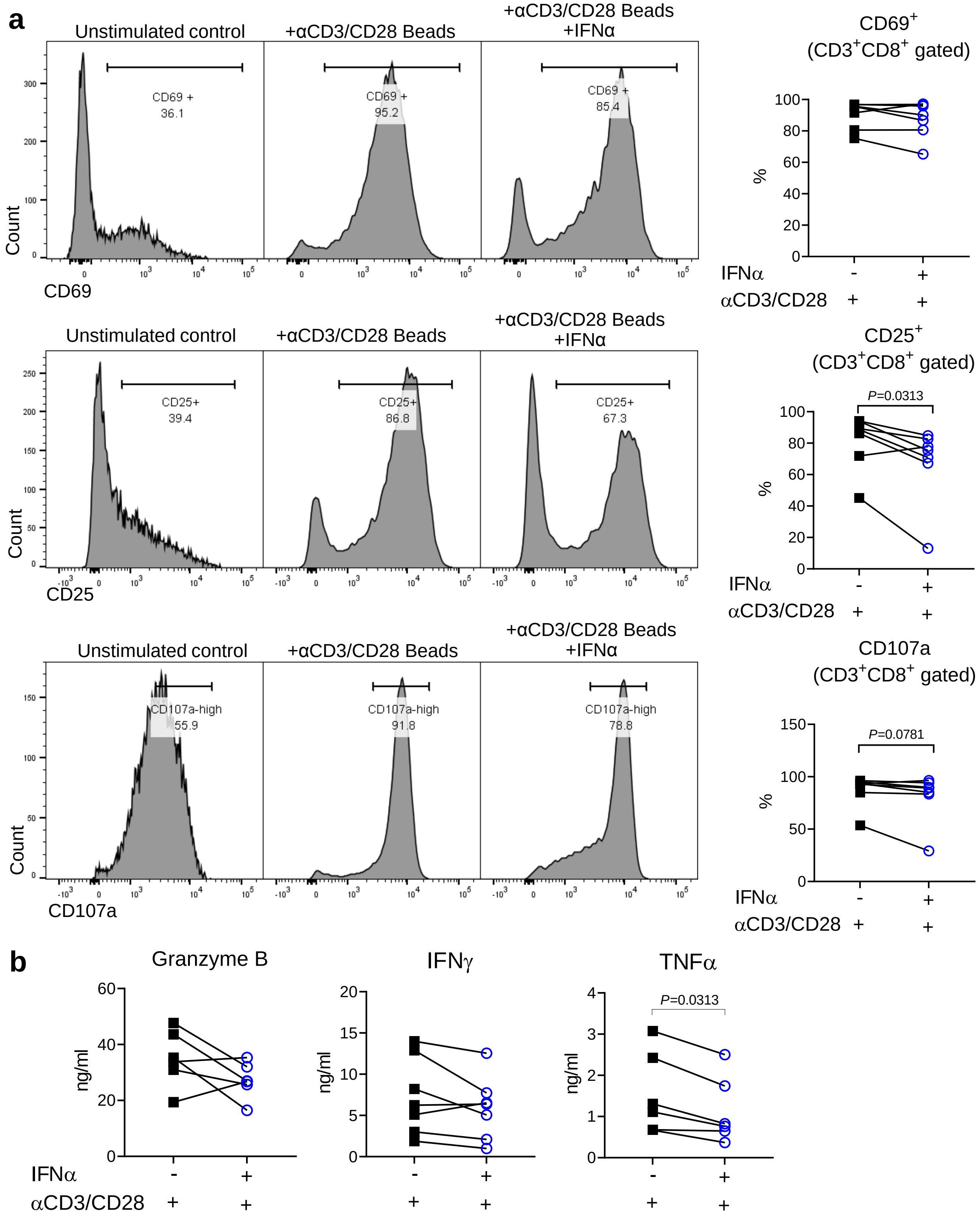


**Supplementary Figure 12** Functional effects of IFNα stimulation on activated CD8+ T cells.

**(a-b)** PBMCs from healthy donors were stimulated with αCD3/CD28 beads in the presence or absence of 1000U/ml of IFNα for 7 days. αCD3/CD28 beads were removed from the culture and cells were rested overnight. CD8+ T cells were then sorted and re-stimulated with αCD3/CD28 beads (beads to cell ratio= 1:2) for 24 hours. Percentage of CD8+ T cells positive for CD69, CD25 and CD107a is shown (n=7). **(b)** Cytokine level in the supernatant measured by ELISA at 24 hours (n=6-7). (**a-b)** Each symbol represents an individual; Two-tailed Wilcoxon matched-pairs signed rank test; only significant differences are indicated. Source data for this figure are provided as a Source Data file

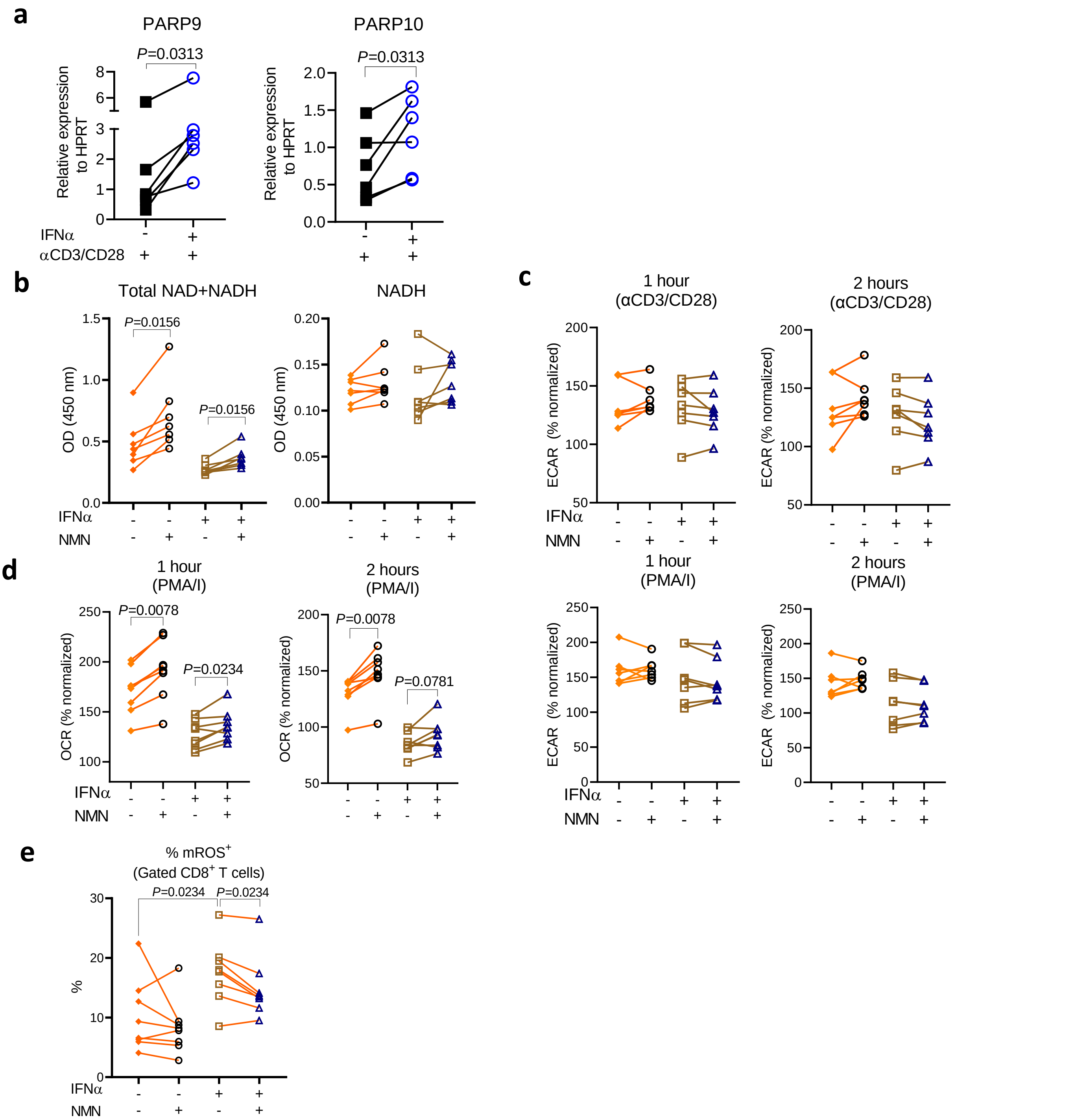


**Supplementary Figure 13**. Downstream effects of NAD+ supplementation on activated CD8$^+$ T cells

Purified CD8$^+$ T cells from healthy donors were treated with IL-2 (10U/ml) and αCD3/CD28 with or without 1000U/ml IFNα for 7 days and with or without addition of 1mM NMN from day 3. At day 7 CD8$^+$ T cells were isolated and rested overnight before further analysis. (**a**) PARP9 and PARP10 gene expression at Day 7 (n=6). **(b)** Total NAD$^+$ and NADH measured using the NAD+/NADH Assay Kit (Abcam). OD values are shown (n=7). (**c-d**) OCR and ECAR levels 1 and 2 hours after re-stimulation with PMA/Ionomycin and αCD3/CD28 beads respectively injected during the extracellular flux assay under the different experimental conditions as indicated. Data were normalized to basal level of each individual (n=8). **(e)** Proportion of CD8$^+$ T cells positive for mROS measured after re-stimulation with αCD3/CD28 (1:2 bead to cell ratio) for 24 hours (n=8). **(a-e)** Data presented as mean ± S.E.M. Each symbol represents one donor. Two-tailed Wilcoxon matched-pairs signed rank test was utilized; only significant data are indicated. Optical Density=OD; β-Nicotinamide mononucleotide=NMN; OCR=oxygen consumption ratio; ECAR= extracellular acidification rate. Source data for this figure are provided as a Source Data file.

**Supplementary Table 1**. Baseline demographic and clinical features of the SLE patients of the transcriptomic study.

| **Age/ Sex** | **Ethnicity** | **LN Class** | **Current Treatment** | **uPCR** (mg/mmol) | **dsDNA** (unit/mL) | **Creatinine** (umol/L) | **Albumin** (g/L) | **SLEDAI** | **BILAG/ Renal** | **SLE (LN) Duration** (Years) | **CD4$^{+}$ T cells** | **CD8$^{+}$ T cells** |
|---|---|---|---|---|---|---|---|---|---|---|---|---|
| 22/F | Caucasian | IV-G (A/C) | MMF, HCQ | 294 | 222 | 69 | 19 | 20 | 28/A | 8 (7) | √ | √ |
| 43/F | Afro-Caribbean | IV-S (A/C) | HCQ, Tac | 431 | 201 | 69 | 26 | 14 | 33/A | 10 (6) | | √ |
| 63/F | Mixed | V | Pred, HCQ | 805 | 28 | 146 | 21 | 6 | 12/A | 31 (21) | | √ |
| 51/F | Afro-Caribbean | V | Pred, HCQ, MMF | 126 | 439 | 64 | 19 | 8 | 13/A | 9 (0) | √ | √ |
| 48/F | Afro-Caribbean | V | HCQ, MMF | 336 | 134 | 104 | 12 | 7 | 24/A | 28 (14) | √ | √ |
| 33/F | Afro-Caribbean | V | MMF, HCQ | 367 | <10 | 70 | 20 | 6 | 13/A | 11 (11) | √ | √ |
| 32/F | Asian | IV-G (A/C) | HCQ, MMF | 119 | 31 | 48 | 24 | 17 | 14/A | 15 (9) | √ | √ |
| 36/F | Caucasian | V | HCQ, MMF | 224 | 105 | 60 | 28 | 9 | 13/A | 7 (5) | | √ |
| 55/F | Afro-Caribbean | V | HCQ, MMF | 260 | <10 | 133 | 34 | 12 | 13/A | 35 (6) | √ | √ |
| 29/F | Asian | IV (A/C) | HCQ, AZA | 150 | 225 | 129 | 26 | 11 | 36/A | 4 (4) | √ | √ |
| 50/F | Afro-Caribbean | III (C) + V | Pred, HCQ, MMF | 107 | 1528 | 61 | 35 | 8 | 17/B | 10 (9) | | √ |
| 53/F | Caucasian | V | Pred | 390 | 54 | 74 | 28 | 8 | 13/A | 0 (0) | √ | √ |
| 48/F | Caucasian | IV-S (A/C) | Pred, HCQ, MMF | 203 | 257 | 82 | 20 | 8 | 13/A | 20 (0) | √ | |
| | | | | | | | | | | | | |
| 26/F | Caucasian | IV-S (C) | HCQ, AZA | 41 | 69 | 63 | 40 | 2 | 0/D | 11 (8) | √ | √ |
| 49/F | Mixed | V | HCQ, MMF | <20 | <10 | 67 | 37 | 0 | 0/D | 12 (4) | √ | √ |
| 54/F | African | IV-S (A) | HCQ, MMF | <20 | <10 | 75 | 33 | 0 | 1/D | 10 (9) | √ | √ |
| 52/F | Arabic | III | None | <20 | <10 | 66 | 42 | 0 | 0/D | 25 (19) | √ | √ |
| 43/F | African | IV | Pred, AZA | <20 | <10 | 89 | 38 | 0 | 0/D | 35 (13) | √ | √ |
| 52/F | Caucasian | IV | Pred | <20 | <10 | 72 | 39 | 0 | 0/D | 35 (28) | √ | √ |
| 66/F | Caucasian | V | HCQ, MMF | 32 | <10 | 63 | 30 | 2 | 1/D | 42 (35) | | √ |
| 33/F | Asian | V | HCQ, MMF | <20 | 11 | 55 | 40 | 0 | 0/D | 8 (8) | √ | √ |
| 56/F | Asian | V | MMF | 34 | 16 | 64 | 39 | 0 | 0/D | 10 (10) | √ | √ |
| 42/F | Caucasian | III (A) | HCQ, AZA | <20 | 239 | 61 | 36 | 4 | 0/D | 15 (15) | √ | √ |
| 36/F | Asian | IV | MMF | <20 | 46 | 52 | 38 | 2 | 2/D | 17 (7) | √ | √ |
| 41/F | Asian | III (A/C) | None | <20 | <10 | 78 | 32 | 0 | 1/D | 12 (11) | √ | √ |
| 39/F | Afro-Caribbean | V+III (A) | HCQ | <20 | 30 | 57 | 35 | 0 | 1/D | 18 (18) | √ | √ |
| 54/F | Asian | V+III (C) | Pred, HCQ, MTX | <20 | <10 | 76 | 34 | 2 | 2/D | 13 (10) | √ | √ |
| 33/F | Afro-Caribbean | IV | HCQ, MMF | <20 | 41 | 72 | 38 | 2 | 0/D | 9 (9) | √ | √ |
| 21/F | Asian | IV | HCQ, MMF | <20 | 23 | 59 | 42 | 1 | 0/D | 5 (5) | √ | √ |

F - female; Albumin (normal range 35-50g/L); BILAG - British Isles Lupus Assessment Group; Creatinine (normal range 55-110umol/L); dsDNA - double stranded DNA (normal range 0-30 unit/mL); LN - lupus nephritis; AZA - azathioprine; HCQ - hydroxychloroquine; MTX - methotrexate; MMF - mycophenolate mofetil; Pred - prednisolone; Tac - tacrolimus; uPCR - urine protein creatinine ratio (normal range <30 mg/nmol); SLEDAI - SLE Disease Activity Index.  LN duration was calculated from the time of the first renal biopsy.

**Supplementary Table 2.** Number of differentially expressed genes (DEGs)

| | Comparisons | Fold-Change > 1.5 | Fold-Change < -1.5 | Total DEGs |
|---|---|---|---|---|
| $CD4^+$ T cells | **All SLE (n=24) vs Healthy Control (n=11)** | **211** | **26** | **237** |
| | Active SLE (n=9) vs Healthy Control (n=11) | 382 | 124 | 506 |
| | Inactive SLE (n=15) vs Healthy Control (n=11) | 70 | 5 | 75 |
| | Active (n=9) vs Inactive SLE (n=15) | 0 | 0 | 0 |
| | SLE-2 (n=13) vs Healthy Control (n=11) | 541 | 239 | 780 |
| | SLE-1 (n=11) vs Healthy Control (n=11) | 58 | 73 | 131 |
| | SLE-2 (n=13) vs SLE-1 (n=11) | 498 | 144 | 642 |
| $CD8^+$ T cells | **All SLE (n=28) vs Healthy Control (n=11)** | **267** | **91** | **358** |
| | Active SLE (n=12) vs Healthy Control (n=11) | 406 | 225 | 631 |
| | Inactive SLE (n=16) vs Healthy Control (n=11) | 95 | 23 | 118 |
| | Active (n=12) vs Inactive SLE (n=16) | 1 | 2 | 3 |
| | SLE-2 (n=15) vs Healthy Control (n=11) | 604 | 238 | 842 |
| | SLE-1 (n=13) vs Healthy Control (n=11) | 21 | 14 | 35 |
| | SLE-2 (n=15) vs SLE-1 (n=13) | 190 | 27 | 217 |

Source data for this table are provided as a Source Data file.

**Supplementary Table 3**. Metabolic and functional studies: baseline demographic and clinical features

| | IFN-High SLE patients (n=25) | IFN-Neg SLE patients (n=20) | Healthy controls (n=24) |
|---|---|---|---|
| Females | 25 (100%) | 20 (100%) | 24 (100%) |
| Males | 0 (0%) | 0 (0%) | 0 (0%) |
| Age (years) | 44 (24-62) | 46 (29-69) | 31 (26-58) |
| Ethnicity: | | | |
| Caucasian | 4 (16%) | 8 (40%) | 13 (54.2%) |
| African | 6 (24%) | 3 (15%) | 1 (4.2%) |
| Asians | 10 (40%) | 3 (15%) | 8 (33.3%) |
| Mixed | 2 (8%) | 4 (20%) | 2 (8.4%) |
| Other Ethnic Groups | 3 (12%) | 2 (10%) | 0 (0%) |
| Medications: | | | |
| Hydroxychloroquine | 19 (76%) | 16 (80%) | n/a |
| Prednisolone | 9 (36%) | 8 (40%) | n/a |
| Mycophenolate Mofetil | 9 (36%) | 9 (45%) | n/a |
| Azathioprine | 2 (8%) | 3 (15%) | n/a |
| No medication | 1 (5.8%) | 2 (10%) | n/a |
| Renal involvement: | 23 (92%) | 18 (90%) | n/a |
| Lupus Nephritis Class III | 7 (28%) | 1 (5%) | n/a |
| Lupus Nephritis Class IV | 10 (40%) | 8 (40%) | n/a |
| Lupus Nephritis Class V | 7 (28%) | 11 (55%) | n/a |

Source data for this table are provided as a Source Data file.

**Supplementary Table 4:** Key resource table for material and methods

| REAGENT or RESOURCE | SOURCE | IDENTIFIER |
|---|---|---|
| **Antibodies** | | |
| Human TruStain FcX™ (Fc Receptor Blocking Solution) – 1:100 dilution | Biolegend | Cat. no. 422302 |
| Tom20 primary antibody – 1:50 dilution | Santa Cruz Biotechnology, Inc | Clone FL-145, cat. no. sc-11415 |
| CD8 alpha primary antibody – 1:200 dilution | Abcam | cat. no. ab199016 |
| Goat anti-rabbit Alexa 488 – 1:200 dilution | Invitrogen | cat. no. A-11008 |
| Goat anti-mouse Alex 568 – 1:200 dilution | Invitrogen | cat. no. A-11004 |
| DAPI (4′,6-diamidino-2-phenylindole) – 1:200 dilution | Thermo Fisher Scientific | cat. no. 62248 |
| Vectashield H-1000 | Vector Labs | cat. no. H-1000-10 |
| CD3 - BV421 (2ul/test) | Biolegend | Clone SK7, cat. no. 344834 |
| CD3 - APC-Cy7 (2ul/test) | Biolegend | Clone HIT3a, cat. no. 300318 |
| CD3 - PE-Cy7 (2ul/test) | Biolegend | Clone UCHT1, cat. no. 300420 |
| CD8 - BV711 (1ul/test) | Biolegend | Clone RPA-T8, cat. no. 301044 |
| CD8 - PE (2ul/test) | Biolegend | Clone SK1, cat. no. 344733 |
| CD8 - APC (2ul/test) | Biolegend | Clone SK1, cat. no. 344706 |
| CD4 - APC (2ul/test) | Biolegend | Clone SK3, cat. no. 344614 |
| CD4 - BV711 (1ul/test) | Biolegend | Clone RPA-T4, cat. no. 300557 |
| CD4 – FITC (2ul/test) | Biolegend | Clone SK3, cat. no. 344604 |
| CCR7 - BV421(2ul/test) | Biolegend | Clone G043H7, cat. no. 353208 |
| CCR7 - BV785 (2ul/test) | Biolegend | Clone G043H7, cat. no. 353230 |
| CD45RA - Percp-cy5.5 (2ul/test) | Biolegend | Clone HI100, cat. no. 304156 |
| CD45RA - PE (1ul/test) | Biolegend | Clone HI100, cat. no. 304108 |
| CD45RA - Alexa Fluor® 488 (2ul/test) | Biolegend | Clone HI100, cat. no. 304114 |
| MT-ND1 – FITC -1:100 dilution | Biorbyt | Cat. no. orb9394 |
| MT-CYTB / Cytochrome b245 – APC - 1:100 dilution | Novus Biotech | Clone CS9, cat. no. NBP1-40974APC |
| MT-APT6 - FITC - 1:100 dilution | Biorbyt | Cat. no. orb189647 |
| Cytochrome C - Alexa Fluor® 647 – 1:200 dilution | Biolegend | Clone 6H2.B4, cat. no. 612310 |
| ATP5O5 - Alexa Fluor® 488 – 1:100 dilution | Abcam | Cat. no. ab198302 |
| CD38 -PE/Cy7 (2ul/test) | Biolegend | Clone HB-7, cat. no. 356608 |
| | | |

| Chemical reagents for mitochondrial staining | | |
|---|---|---|
| Mitotracker Green (MTG), 50nM | Thermo Fisher Scientific | M7514 |
| Mitotracker Deep Red (MTDR), 10nM | Thermo Fisher Scientific | M22426 |
| Tetramethylrhodamine (TMRM), 25 nM | Thermo Fisher Scientific | T668 |
| MitoSOX Red, 25nM | Thermo Fisher Scientific | M36008 |
| CellROX Deep Red, 2.5uM | Thermo Fisher Scientific | C10422 |
| | | |
| **Biological Samples** | | |
| Buffy coat cones | NHSBT | NC24 |
| | | |
| **Chemicals, Peptides, and Recombinant Proteins** | | |
| Lymphoprep | STEMCELL Technologies | 7851 |
| Pan T Cell Isolation Kit | Miltenyi Biotec | 130-096-535 |
| Dynabeads FlowComp Human CD4 Kit | Invitrogen | 11361D |
| Dynabeads FlowComp Human CD8 Kit | Invitrogen | 11362D |
| Human CD8+ T Cell Isolation Kit | Myltenyi Biotec | 130-096-495 |
| RNeasy Mini kits | Qiagen | 74104 |
| RNase-free DNase Kit | Qiagen | 79254 |
| TruSeq Stranded mRNA HT kit | Illumina, UK | 20020594 |
| AMPure XP Beads | Beckman Coulter | A63880 |
| iScript cDNA Synthesis Kit | Bio-Rad, USA | 1708891 |
| Power SYBR Green Master Mix | Applied Biosystems, USA | 4364346 |
| Universal Type I IFN Protein | R&D System | 11200-2 |
| Recombinant Human IL-2 | Peprotech | 200-02 |
| Seahorse XF RPMI medium | Agilent | 103576-100 |
| Seahorse XF Cell Mito Stress Test Kit | Agilent | 103015-100 |
| Dynabeads Human T-Activator CD3/CD28 (1:4 beads to cell ratio) | Thermo Fisher Scientific | 11131D |
| Live/dead Fixable Aqua stain kit | Life Technologies | L34966 |
| Fixation/permeabilization solution | BD Biosciences | 554714 |
| XF Cell Mito Stress kit | Agilent Technologies, USA | 103015-100 |
| Cell-Tak Cell and Tissue Adhesive | Corning, USA | 354240 1 |
| Seahorse XF RPMI medium | Agilent Technologies, USA | 103576-100 |
| L-Glutamine | Sigma | G7513-100ML |
| Pyruvate | S8636-100ML | Thermo Fisher Scientific |

| | | |
|---|---|---|
| Glucose | sigma | D6134-1G |
| Oligomycin | Sigma | O4876-5MG |
| Fluorocarbonylcyanide phenylhydrazone (FCCP) | Sigma | C2920-10MG |
| Rotenone | Sigma | R8875-5G |
| Antimycin A | Sigma | A8674-25MG |
| phorbol12-myristate13-acetate (PMA) / Ionomycin | Sigma/ MerckMillipore | P8139 5mg/ 407950-1MG |
| CFSE CellTrace™ CFSE Cell Proliferation Kit, for flow cytometry | Thermo Fisher Scientific | C34554 |
| RPMI 1640, no glutamine (500ml) | Invitrogen | 31870025 |
| β-Nicotinamide mononucleotide | Sigma-Aldrich | N3501 |
| NAD/NADH Assay Kit (Colorimetric) | Abcam | ab65348 |
| 10kD Spin Column | Abcam | ab98849 |
| | | |
| **Critical Commercial Assays** | | |
| Human TNF-alpha DuoSet ELISA | R&D System | DY210-05 |
| Human IFN-gamma DuoSet ELISA | R&D System | DY285B-05 |
| Human Granzyme B DuoSet ELISA | R&D System | DY2906-05 |
| Bioluminescent ATP assay kit | Abcam | ab113849 |
| FITC-Annexin V Apoptosis Detection Kit | BD Bioscience | 556420 |
| V450-Annexin V Apoptosis Detection Kit | BD Bioscience | 560506 |
| | | |
| **Deposited Data** | | |
| GSE97263 | https://www.ncbi.nlm.nih.gov/geo/query/acc.cgi?acc=GSE97263 | |
| GSE97264 | https://www.ncbi.nlm.nih.gov/geo/query/acc.cgi?acc=GSE97264 | |
| | | |
| **Oligonucleotides** | **Forward Sequence** | **Reverse Sequence** |
| mtDNA-encoded tRNA | CACCCAAGAACAGGGTTTGT | TGGCCATGGGTATGTTGTTA |
| nDNA-encoded B2M | TGCTGTCTCCATGTTTGATGTATCT | TCTCTGCTCCCCACCTCTAAGT |
| 18s | CCGCAGCTAGGAATAATGGA | ACCTCCGACTTTCGTTCTTG |
| CMPK2 | TGACCTTATCCTGCTGCTCA | CGTCTGCAGGACCTTTTCTC |

| | | |
|---|---|---|
| HERC5 | TCATTCTCCACCCCAAGAAG | CATCTGGACCAGTTTGCTGA |
| ESPTI | CCAGACAGAAGTGCCTGTCA | TCTGGTGGATTTTGGCTCTT |
| MT-CYTB | TATCCGCCATCCCATACATT | TTTTATCGGAATGGAGGTG |
| MT-ATP6 | GCCCTAGCCCACTTCTTACC | GTGGCGCTTCCAATTAGGTG |
| MT-ND1 | CTACTACAACCCTTCGCTGAC | GGATTGAGTAAACGGCTAGGC |
| MT-ND3 | TGCGGCTTCGACCCTATATC | GCCAGACTTAGGGCTAGGAT |
| HPRT | TGAGGATTTGGAAAGGGTGT | AATCCAGCAGGTCAGCAAAG |
| COX4L1 | Taqman probe: Hs00971639_m1 | |
| SDHAF4 | Taqman probe: Hs00540918_m1 | |
| | | |
| **Software and Algorithms** | | |
| R Studio (v.1.4.1103) | https://rstudio.com/products/rstudio | |
| ggplot2 (v.3.0.0) | https://cran.r-project.org/web/packages/ggplot2/index.html | |
| pheatmap (v 1.0.10) | https://cran.r-project.org/web/packages/pheatmap/index.html | |
| pcaExplorer (v.2.6.0) | http://bioconductor.riken.jp/packages/3.7/bioc/vignettes/pcaExplorer/inst/doc/pcaExplorer.html | |
| DESeq2 (v.1.14.1) | https://bioconductor.org/packages/release/bioc/html/DESeq2.html | |
| edgeR (v.3.22.3) | https://bioconductor.org/packages/release/bioc/html/edgeR.html | |
| Rsubread (v.1.30.5) | https://bioconductor.org/packages/release/bioc/html/Rsubread.html | |
| HT-Seq-count (v.0.6.1) | https://htseq.readthedocs.io/en/master/ | |
| picard (v.2.6.0) | https://broadinstitute.github.io/picard/ | |
| FastQC (v.0.11.2) | https://www.bioinformatics.babraham.ac.uk/projects/fastqc/ | |
| Trimmomatic (v.0.36) | http://www.usadellab.org/cms/?page=trimmomatic | |
| tophat2 (v.2.1.0) | https://ccb.jhu.edu/software/tophat/index.shtml | |
| Cytoscape ClueGo v.2.3.3 | http://apps.cytoscape.org/apps/cluego | |
| Cytoscape (V3.6.0) | https://cytoscape.org/ | |
| GSVA v1.30.0 | https://www.bioconductor.org/packages/release/bioc/html/GSVA.html | |
| WGCNA v.1.68 | https://bmcbioinformatics.biomedcentral.com/articles/10.1186/1471-2105-9-559 | |
| STRING v.11 | https://string-db.org/ | |
| FlowJo software, version 10 | Tree Star Inc. Ashland, OR, USA | |
| Imaris 9.5.1 software (version 9.5.1) | Bitplane AG | |
| GraphPad Software v.9.0.1 | Inc. La Jolla California, USA | |

2

Brilliant VioletTM (BV); Allophycocyanin (APC); Allophycocyanin-Cyanine7 (APC-Cy7); Phycoerythrin (PE); Phycoerythrin-Cyanine7 (PE-Cy7) Inc. La Jolla California, USA; fluorescein isothiocyanate (FITC)
