# Supplementary material for: Type I interferons affect the metabolic fitness of CD8+ T cells from patients with systemic lupus erythematosus
Source: Nat Commun. 2021 Mar 31;12:1980. doi: 10.1038/s41467-021-22312-y (PMC8012390; doi:10.1038/s41467-021-22312-y)
Supplement: Supplementary file 3 — Reporting Summary [file 41467_2021_22312_MOESM3_ESM.pdf]

nature research

Corresponding author(s): Marina Botto

Last updated by author(s): Feb 20, 2021

Reporting Summary

Nature Research wishes to improve the reproducibility of the work that we publish. This form provides structure for consistency and transparency in reporting. For further information on Nature Research policies, see our Editorial Policies and the Editorial Policy Checklist.

Statistics

For all statistical analyses, confirm that the following items are present in the figure legend, table legend, main text, or Methods section.

n/a	Confirmed	
☐	☒	The exact sample size (*n*) for each experimental group/condition, given as a discrete number and unit of measurement
☐	☒	A statement on whether measurements were taken from distinct samples or whether the same sample was measured repeatedly
☐	☒	The statistical test(s) used AND whether they are one- or two-sided<br>*Only common tests should be described solely by name; describe more complex techniques in the Methods section.*
☐	☒	A description of all covariates tested
☐	☒	A description of any assumptions or corrections, such as tests of normality and adjustment for multiple comparisons
☐	☒	A full description of the statistical parameters including central tendency (e.g. means) or other basic estimates (e.g. regression coefficient) AND variation (e.g. standard deviation) or associated estimates of uncertainty (e.g. confidence intervals)
☐	☒	For null hypothesis testing, the test statistic (e.g. *F*, *t*, *r*) with confidence intervals, effect sizes, degrees of freedom and *P* value noted<br>*Give P values as exact values whenever suitable.*
☒	☐	For Bayesian analysis, information on the choice of priors and Markov chain Monte Carlo settings
☐	☒	For hierarchical and complex designs, identification of the appropriate level for tests and full reporting of outcomes
☐	☒	Estimates of effect sizes (e.g. Cohen's *d*, Pearson's *r*), indicating how they were calculated

Our web collection on statistics for biologists contains articles on many of the points above.

Software and code

Policy information about availability of computer code

Data collection	Flow cytometry data were collected using LSRFortessa flow cytometer (BD Biosciences, USA).<br><br>For GSVA and WGCNA analysis, a priori defined gene set was manually downloaded from MSig database (http://software.broadinstitute.org/gsea/msigdb).<br><br>The dataset from this study have been deposited in the Gene Expression Omnibus repository (GEO series accession number: GSE97263 [https://www.ncbi.nlm.nih.gov/geo/query/acc.cgi?acc=GSE97263] and GSE97264 [https://www.ncbi.nlm.nih.gov/geo/query/acc.cgi?acc=GSE97264]). All data are available in the main text or the supplementary materials. Raw data is available from the authors and Source Data File.
Data analysis	Software and Algorithms:<br><br>R Studio v.1.4.1103 for Windows<br><br>Following softwares were used for RNA-seq analysis: ggplot2 (v.3.0.0, https://cran.r-project.org/web/packages/ggplot2/index.html), pheatmap (v 1.0.10, https://cran.r-project.org/web/packages/pheatmap/index.html), pcaExplorer (v.2.6.0, http://bioconductor.riken.jp/packages/3.7/bioc/vignettes/pcaExplorer/inst/doc/pcaExplorer.html), DESeq2 (v.1.14.1, https://bioconductor.org/packages/release/bioc/html/DESeq2.html), edgeR (v.3.22.3, https://bioconductor.org/packages/release/bioc/html/edgeR.html), Rsubread (v.1.30.5, https://bioconductor.org/packages/release/bioc/html/Rsubread.html); HT-Seq-count (v.0.6.1, https://htseq.readthedocs.io/en/master/), picard (v.2.6.0, https://broadinstitute.github.io/picard/), FastQC (v.0.11.2, https://www.bioinformatics.babraham.ac.uk/projects/fastqc/), Trimmomatic (v.0.36, http://www.usadellab.org/cms/?page=trimmomatic), tophat2 (v.2.1.0, https://ccb.jhu.edu/software/tophat/index.shtml)

Transcriptomic pathway analysis was done using following softwares: Cytoscape ClueGo (v.2.3.3, http://apps.cytoscape.org/apps/cluego), Cytoscape (V3.6.0, https://cytoscape.org/), GSVA (v1.30.0 https://www.bioconductor.org/packages/release/bioc/html/GSVA.html), WGCNA (v.1.68, https://bmcbioinformatics.biomedcentral.com/articles/10.1186/1471-2105-9-559), STRING (v.11, https://string-db.org/).

Flow cytometry analysis was done using FlowJo software (version 10 Tree Star Inc. Ashland, OR, USA).

Mitochondrial imaging analysis was done using Imaris 9.5.1 software (version 9.5.1, Bitplane AG),

Statistical analysis was done using GraphPad Software (v.9.0.1, Inc. La Jolla California, USA)

For manuscripts utilizing custom algorithms or software that are central to the research but not yet described in published literature, software must be made available to editors and reviewers. We strongly encourage code deposition in a community repository (e.g. GitHub). See the Nature Research guidelines for submitting code & software for further information.

Data

Policy information about availability of data

All manuscripts must include a data availability statement. This statement should provide the following information, where applicable:

- Accession codes, unique identifiers, or web links for publicly available datasets
- A list of figures that have associated raw data
- A description of any restrictions on data availability

The dataset from this study have been deposited in the Gene Expression Omnibus repository (GEO series accession number: GSE97263 [https://www.ncbi.nlm.nih.gov/geo/query/acc.cgi?acc=GSE97263] and GSE97264 [https://www.ncbi.nlm.nih.gov/geo/query/acc.cgi?acc=GSE97264])

For GSVA and WGCNA analyses, a priori defined gene sets from BIOCARTA, KEGG, and REACTOME databases were derived from MSig database (http://software.broadinstitute.org/gsea/msigdb).

Microscopy image datasets are available from the corresponding author upon request.

All the other data are available in the main text or the supplementary materials. The raw data that support all the remaining figures/tables are provided in the Data Source File.

Field-specific reporting

Please select the one below that is the best fit for your research. If you are not sure, read the appropriate sections before making your selection.

☒ Life sciences ☐ Behavioural & social sciences ☐ Ecological, evolutionary & environmental sciences

For a reference copy of the document with all sections, see nature.com/documents/nr-reporting-summary-flat.pdf

Life sciences study design

All studies must disclose on these points even when the disclosure is negative.

Sample size	the sample size for transcriptomic analysis were based on a previously published study suggesting minimum of 10 sample size per group to achieve 70% power using various power calculation methods for RNA-seq data (PROPER, Scotty, SSPA, RNAseqPowerCalculator; https://doi.org/10.1093/bib/bbw144).<br><br>For functional studies, size was determined using power calculation where appropriate.
Data exclusions	The dataset from this study have been deposited in the Gene Expression Omnibus repository (GEO series accession number: GSE97263 [https://www.ncbi.nlm.nih.gov/geo/query/acc.cgi?acc=GSE97263] and GSE97264 [https://www.ncbi.nlm.nih.gov/geo/query/acc.cgi?acc=GSE97264]).<br><br>Transcriptomic data from Active SLE (CDR2, CDR20, CDR36, CDR65), Inactive SLE (CDR50), and Healthy (LHC-7, LHC-8, LHC-15) were not included in the final analysis. These samples are from male patients and as we found significant differences in the transcriptome of male vs female SLE patients. Thus we focused our analysis only on female SLE patients which is the large majority of our data. Transcriptomic data from Inactive patient CDR54 was also excluded from the final analysis as this patient was later found to have a different renal pathology.
Replication	All functional experiments were repeated at least once and similar results were observed in all repeated experiments. The final data shown in the paper are the combination of all the experiments.
Randomization	Randomization was not relevant for our studies.<br><br>For transcriptomic study, we selected twenty-nine biopsy-proven Lupus Nephritis (LN) patients recruited from the Imperial Lupus Centre and the details are presented in Supplementary Table 1. Eleven healthy female volunteers (with no family history of autoimmune disease) served as age- and ethnicity-matched controls.

For the metabolic and functional studies, we selected inactive lupus patients according to their IFN signature and their demographics are summarised in Supplementary Table 3. Seven female patients with Rheumatoid Arthritis (RA) were recruited as disease controls for the metabolic study.

Blinding

Imaging analysis was done in a blinded manner by different researchers from those preparing the slides. Patient selection was not blinded as patients were selected according to their IFN signature. For other experiments, blinding during data collection and/or analysis was not possible, as the same researchers were involved in sample collection, functional experiments, and data analysis. The researchers need to know the patient group to conduct these studies but the data were based on objectively measurable results.

Reporting for specific materials, systems and methods

We require information from authors about some types of materials, experimental systems and methods used in many studies. Here, indicate whether each material, system or method listed is relevant to your study. If you are not sure if a list item applies to your research, read the appropriate section before selecting a response.

Materials & experimental systems

n/a	Involved in the study	
☐	☒	Antibodies
☒	☐	Eukaryotic cell lines
☒	☐	Palaeontology and archaeology
☒	☐	Animals and other organisms
☐	☒	Human research participants
☒	☐	Clinical data
☒	☐	Dual use research of concern

Methods

n/a	Involved in the study	
☒	☐	ChIP-seq
☐	☒	Flow cytometry
☒	☐	MRI-based neuroimaging

Antibodies

Antibodies used

Tom20 primary antibody Santa Cruz Biotechnology, Inc FL-145, (Clone FL-145, Cat. no sc-11415, 1:50 dilution, https://www.scbt.com/p/tom20-antibody-fl-145?requestFrom=search)
CD8 alpha primary antibody, Abcam, (Cat. no ab199016, 1:200 dilution, https://www.abcam.com/cd8-alpha-antibody-c8468--c8144b-ab199016.html)
Goat anti-rabbit Alexa 488, Invitrogen, (Cat. no. A-11008, 1:200 dilution, https://www.thermofisher.com/antibody/product/Goat-anti-Rabbit-IgG-H-L-Cross-Adsorbed-Secondary-Antibody-Polyclonal/A-11008)
Goat anti-mouse Alex 568 Invitrogen, (Cat. no A-11004, 1:200 dilution, https://www.thermofisher.com/antibody/product/Goat-anti-Mouse-IgG-H-L-Cross-Adsorbed-Secondary-Antibody-Polyclonal/A-11004)
DAPI (4′,6-diamidino-2-phenylindole), Thermo Fisher Scientific, (Cat. no 62248, 1:200 dilution, https://www.thermofisher.com/order/catalog/product/62248?SID=srch-hj-62248#/62248?SID=srch-hj-62248)
CD3 - BV421, Biolegend, (SK7, cat. no. 344834, 2ul/test, https://www.biolegend.com/en-us/products/brilliant-violet-421-anti-human-cd3-antibody-11839)
CD3 - APC-Cy7, Biolegend, (HIT3a, cat. no. 300318, 2ul/test, https://www.biolegend.com/en-us/products/apc-cyanine7-anti-human-cd3-antibody-1912)
CD3 - PE-Cy7, Biolegend, (UCHT1, cat. no. 300420, 2ul/test, https://www.biolegend.com/en-us/products/pe-cyanine7-anti-human-cd3-antibody-3070)
CD8 - BV711, Biolegend, (RPA-T8, cat. no. 301044, 1ul/test, https://www.biolegend.com/en-us/products/brilliant-violet-711-anti-human-cd8a-antibody-7929)
CD8 - PE, Biolegend, (SK1, cat. no. 344733, 2ul/test, https://www.biolegend.com/en-us/products/brilliant-violet-711-anti-human-cd8-antibody-10762)
CD8 - APC, Biolegend, (SK1, cat. no. 344706, 2ul/test, https://www.biolegend.com/en-us/products/pe-anti-human-cd8-antibody-6247)
CD4 - APC, Biolegend, (SK3, cat. no. 300557, 1ul/test, https://www.biolegend.com/en-us/products/brilliant-violet-711-anti-human-cd4-antibody-10435)
CD4 - BV711, Biolegend, (RPA-T4, cat. no. 300557, 1ul/test, https://www.biolegend.com/en-us/products/brilliant-violet-711-anti-human-cd4-antibody-10435)
CD4 - FITC, Biolegend, (SK3, cat. no. 344604, 2ul/test, https://www.biolegend.com/en-us/products/fitc-anti-human-cd4-antibody-6206)
CCR7 - BV421, Biolegend, (G043H7, cat. no. 353208, 2ul/test, https://www.biolegend.com/en-us/products/brilliant-violet-421-anti-human-cd197-ccr7-antibody-7497)
CCR7 - BV785, Biolegend, (G043H7, cat. no. 353230, 2ul/test, https://www.biolegend.com/en-us/products/brilliant-violet-785-anti-human-cd197-ccr7-antibody-7983)
CD45RA - Percp-cy5.5, Biolegend, (HI100, cat. no. 304156, 2ul/test, https://www.biolegend.com/en-us/products/percp-anti-human-cd45ra-antibody-13358)
CD45RA - PE, Biolegend,(HI100, cat. no. 304108, 1ul/test, https://www.biolegend.com/en-us/products/pe-anti-human-cd45ra-antibody-687)
CD45RA - Alexa Fluor® 488, Biolegend, (HI100, cat. no. 304114, 2ul/test, https://www.biolegend.com/en-us/products/alexa-fluor-488-anti-human-cd45ra-antibody-3337)
MT-ND1 - FITC Biorbyt, (cat no. orb9394, 1:100 dilution, https://www.biorbyt.com/mt-nd1-antibody-fitc-orb9394.html)
MT-CYTB / Cytochrome b245 - APC, Novus Biotech, (Cat. no. CS9, cat. no. NBP1-40974APC, 1:100 dilution, https://

www.novusbio.com/products/cytochrome-b245-alpha-antibody-cs9_nbp1-40974apc)
MT-APT6 - FITC, Biorbyt, (cat. no. orb189647, 1:100 dilution, https://www.biorbyt.com/mt-atp6-antibody-fitc-orb189647.html)
Cytochrome C - Alexa Fluor® 647, Biolegend, (clone 6H2.B4, cat. no. 612310, 1:200 dilution, https://www.biolegend.com/en-us/products/alexa-fluor-647-anti-cytochrome-c-antibody-10789)
ATP5O5 - Alexa Fluor® 488, Abcam, (cat. no. ab198302, 1:100 dilution, https://www.abcam.com/alexa-fluor-488-atp5o-antibody-4c11c10d12-ab198302.html)
CD38 -PE/Cy7 - Biolegend (HB-7, cat. no. 356608, 2ul/test, https://www.biolegend.com/en-us/products/pe-cyanine7-anti-human-cd38-antibody-8420)

Validation

All antibodies used have been validated by the manufacturer and used according to the manufacturer's instruction. The manufacturer's websites stating the validation of each antibody are listed above. Antibodies have been re-validated by titrating their concentration. Isotypes and FMO were included in the experiments whenever appropriate.

Human research participants

Policy information about studies involving human research participants

Population characteristics

All the lupus patients in the study met the revised American College of Rheumatology criteria and the SLICC criteria and had biopsy-proven nephritis. Lupus nephritis subsets were categorized according to the International Society of Nephrology/Renal Pathology Society classification. Patients who had received Cyclophosphamide and/or B-cell depletion within 6 months were excluded. For the transcriptomic study twenty-nine biopsy-proven Lupus Nephritis (LN) patients were recruited from the Imperial Lupus Centre and the details are presented in Supplementary Table 1. The Systemic Lupus Erythematosus Disease Activity Index (SLEDAI score) and British Isles Lupus Assessment Group (BILAG) index were used for clinical classification; active disease was defined as a SLEDAI > 6 and inactive SLEDAI < 4. Eleven healthy female volunteers (with no family history of autoimmune disease) served as age- and ethnicity-matched controls. The details of the SLE cohort and healthy volunteers involved in the metabolic and functional studies are summarised in Supplementary Table 3. Seven female patients with Rheumatoid Arthritis (RA) were recruited as disease controls for the metabolic study.

Recruitment

Patients who match the above characteristic were approached and recruited into the studies without any additional bias.

Ethics oversight

All subjects gave informed consent and samples were collected as a sub-collection registered with the Imperial College Healthcare Tissue Bank (licence: 12275; National Research Ethics Service approval 17/WA/0161). The Tissue Management Committee of the Imperial College Healthcare Tissue Bank approved the study (ref: R13010a).

Note that full information on the approval of the study protocol must also be provided in the manuscript.

Flow Cytometry

Plots

Confirm that:

- [x] The axis labels state the marker and fluorochrome used (e.g. CD4-FITC).
- [x] The axis scales are clearly visible. Include numbers along axes only for bottom left plot of group (a 'group' is an analysis of identical markers).
- [x] All plots are contour plots with outliers or pseudocolor plots.
- [x] A numerical value for number of cells or percentage (with statistics) is provided.

Methodology

Sample preparation

PBMC separation and T cell isolation

Peripheral blood mononuclear cells (PBMCs) were obtained by density gradient centrifugation using Lymphoprep (STEMCELL Technologies, Canada). In short, approximately 50 ml of blood were diluted 1x with phosphate buffered saline (PBS) with addition of 2% FBS and layered on top of 15 ml of Lymphoprep solution. The sample was then centrifuged at 800 g for 20 minutes at room temperature without break. PBMCs were collected from the interface and washed twice with PBS/2%FBS. To maximize the purity of CD4+ and CD8+ T cells, T cells were first enriched using negative magnetic selection Pan T Cell Isolation Kit (Miltenyi Biotec) per manufacturer's instruction. For metabolic studies, as indicated, the negatively selected T cells were then labelled with anti-CD4 and -CD8 antibodies and sorted using Aria II FACS (Becton-Dickson). For RNA sequencing experiments, CD4+ and CD8+ T cells were positively isolated using Dynabeads FlowComp Human CD4 or CD8 Kit (Invitrogen) according to the manufacturer's protocol. The average purity as assessed by flow cytometry was more than 95%.

For some in vitro culture experiments CD8+ T cells were isolated from buffy coat cones (NHSBT) from healthy donors. CD8+ T cells were purified using human CD8+ T Cell Isolation Kit (Myltenyi Biotec) according to the manufacturer's protocol. Cells were centrifuged and resuspended in complete medium (CM) containing RPMI 1640, 10% of heat-inactivated FBS, 1% of L-glutamine with penicillin-streptomycin, 0.1% of 50 mM β-mercaptoethanol, 2% of 1M HEPES buffer solution, 1% of MEM non-essential amino acids solution and 2% of 100 mM Sodium pyruvate solution. The total number of cells was counted by trypan blue staining with hematocytometer. The average purity as assessed by flow cytometry was more than 85%.

Flow cytometry staining

To exclude dead cells from staining, Live/dead Fixable Aqua stain kit (Molecular Probes, Life Technologies) was used

	according to the manufacturer's instructions. Staining was performed in the presence of a saturating concentration of 2.4G2 monoclonal antibody (anti-CD16/32). For intracellular protein staining, cells were incubated in 1X of BD Fix/Perm™ buffer (BD Biosciences) for 20 minutes at 4°C. Cells were washed with 1X of BD Perm/Wash™ buffer (BD Biosciences) and incubated with antibodies for 30 minutes. After washing with BD Perm/Wash™ buffer, cells were resuspended in FACs buffer (DPBS supplemented with 0.1% BSA, 2mM EDTA and 0.09% Sodium azide). Mitochondrial phenotype was assessed using mitochondrial specific fluorescent probes (Supplementary Table 4). 5 x 105 cells were suspended in 200ul of CM and treated with mitochondrial probes at indicated concentrations. Cells were incubated for 30 minutes at 37°C in a humidified atmosphere containing 5% CO2 before harvesting for flow cytometry analysis.
Instrument	LSRFortessa flow cytometer (BD Biosciences, USA)<br><br>Aria II FACS (Becton-Dickson) for FACs sorting
Software	FlowJo software, version 10 (Tree Star Inc. Ashland, OR, USA)
Cell population abundance	T cell purity isolated using negative selection (Myltenyi Biotec) was more than 85% while positive selection using Dynabeads FlowComp Human CD4 or CD8 Kit (Invitrogen) was more than 95%. After FA sorting the purity of samples are 99%.
Gating strategy	Each relevant gating strategy was mentioned in the figure legends or showed in the figures. Prior to gating cell population of interest, cell debris was removed based on FCS/SSC and only live cell (BV510-Aqua negative) populations were analyzed.<br><br>Supplementary Figure 5 shows gating strategies for CD4+ and CD8+ T cell subsets, mROS, and cROS positive cells. Data in Figure 2 (a-d), Supplementary Figure 6 and 7 were analyzed using these gating strategies. Representative FACs plot for CD69 +, CD25+, and CD107a-high cells are included in Supplementary Figure 12a. Gating strategies for Ann V/PI staining was shown in Figure 3b and CFSE staining was showed in Figure 5b.

☒ Tick this box to confirm that a figure exemplifying the gating strategy is provided in the Supplementary Information.
